# Binding of longer Aβ to transmembrane domain 1 of presenilin 1 impacts on Aβ42 generation

**DOI:** 10.1186/1750-1326-9-7

**Published:** 2014-01-13

**Authors:** Yu Ohki, Naoaki Shimada, Aya Tominaga, Satoko Osawa, Takuya Higo, Satoshi Yokoshima, Tohru Fukuyama, Taisuke Tomita, Takeshi Iwatsubo

**Affiliations:** 1 Department of Neuropathology and Neuroscience, Graduate School of Pharmaceutical Sciences, The University of Tokyo, 7-3-1 Hongo, Bunkyo-ku, Tokyo 113-0033, Japan; 2 Department of Synthetic Natural Chemistry, Graduate School of Pharmaceutical Sciences, The University of Tokyo, Bunkyo-ku, Tokyo 113-0033, Japan; 3 Laboratory of Natural Products Chemistry, Graduate School of Pharmaceutical Sciences, Nagoya University, Nagoya 464-8601, Japan; 4 Core Research for Evolutional Science and Technology, Japan Science and Technology Agency, Bunkyo-ku, Tokyo 113-0033, Japan; 5 Department of Neuropathology, Graduate School of Medicine, The University of Tokyo, Bunkyo-ku, Tokyo 113-0033, Japan

**Keywords:** Presenilin, Secretases, Alzheimer disease, Intramembrane proteolysis, γ-Secretase modulator

## Abstract

**Background:**

Amyloid-β peptide ending at 42nd residue (Aβ42) is believed as a pathogenic peptide for Alzheimer disease. Although γ-secretase is a responsible protease to generate Aβ through a processive cleavage, the proteolytic mechanism of γ-secretase at molecular level is poorly understood.

**Results:**

We found that the transmembrane domain (TMD) 1 of presenilin (PS) 1, a catalytic subunit for the γ-secretase, as a key modulatory domain for Aβ42 production. Aβ42-lowering and -raising γ-secretase modulators (GSMs) directly targeted TMD1 of PS1 and affected its structure. A point mutation in TMD1 caused an aberrant secretion of longer Aβ species including Aβ45 that are the precursor of Aβ42. We further found that the helical surface of TMD1 is involved in the binding of Aβ45/48 and that the binding was altered by GSMs as well as TMD1 mutation.

**Conclusions:**

Binding between PS1 TMD1 and longer Aβ is critical for Aβ42 production.

## Background

Several lines of evidence suggest that the accumulation of amyloid-β peptide (Aβ), a major component of senile plaques, is a common pathological feature in Alzheimer disease (AD) [1]. Aβ is generated through sequential cleavage by β- and γ-secretases of amyloid-β precursor protein (APP). γ-Secretase primarily cleaves APP to produce a C-terminal stub of APP (APP-CTF). Then, scission of APP-CTF by γ-secretase results in generation of various forms of Aβ with different C-terminal lengths. Especially, Aβ ending at the 42nd residue (Aβ42), the most aggregable species, is initially and predominantly deposited in AD brains [2]. Moreover, familial AD-linked mutations in *Psen* (*Presenilin; PS*) *1*, *Psen2* or *APP* genes cause an increase in Aβ42 generation. Thus, Aβ42 is considered as the most pathogenic species causative for AD [3].

γ-Secretase is an intramembrane-cleaving protease complex composed of four membrane spanning proteins: PS, Nicastrin, Aph-1 and Pen-2 [4,5]. Extensive biochemical studies showed that the γ-secretase-mediated intramembrane cleavage of APP occurs in a processive manner [6]; APP-CTF is primarily cleaved at the ϵ-site located around the membrane-cytoplasm boundary to produce Aβ49 or Aβ48. Subsequently, these longer Aβ peptides are processed by stepwise cleavages to secrete shorter Aβ in two predominant production lines: Aβ49 is processed to Aβ43/40 via Aβ46 (Aβ40 production line), and Aβ48 is processed to Aβ42/38 via Aβ45 (Aβ42 production line). PS forms a channel-like catalytic pore structure within the membrane, and is endoproteolyzed to generate N- and C-terminal fragments (NTF and CTF, respectively) during the assembly of the protease-active complex [7,8].

Recently, small compounds that selectively regulate Aβ42 production without affecting ϵ-cleavage emerged, which are termed γ-secretase modulators (GSMs) [9]. We have shown that a potent Aβ42-lowering compound, GSM-1, directly targets the PS1 TMD1 [10]. Moreover, using substituted cysteine accessibility method (SCAM), we identified two different regions within TMD1 of PS1, i.e., a hydrophobic luminal region and a hydrophilic portion facing the catalytic site [11], that are differently involved in the action of GSM-1 [10]. However, the precise molecular mechanism whereby γ-secretase generates Aβ42, as well as the role of TMD1 in Aβ42 production, remains elusive. In this study, we identified TMD1 of PS1 as a regulatory domain for the processive cleavage of the Aβ42 production line.

## Results

### Fenofibrate directly targets the N-terminal fragment of presenilin 1

**Figure 1:**
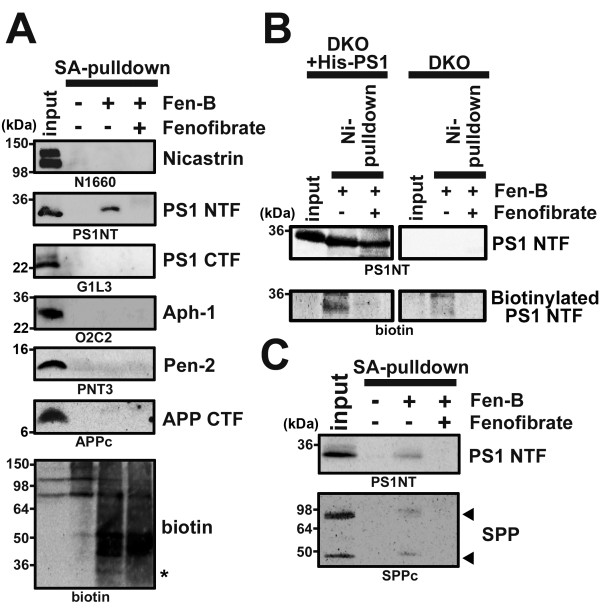
**Fen-B directly targets PS1 and SPP. (A)** PAL experiments using mouse brain membranes by Fen-B (10 μM). This labeling was competed by treatment with fenofibrate (200 μM). Note that only PS1 NTF was specifically labeled by Fen-B. Anti-biotin antibody also reacted with a band with ~30 kDa corresponding to PS1 NTF (an asterisk). **(B)** Fen-B labeling experiment for the DKO cells expressing His-tagged PS1. After PAL, His-tagged PS1 was purified with Ni^2+^-affinity column and analyzed by immunoblotting. **(C)** Fen-B also bound endogenous monomeric as well as dimeric SPP (arrows) in CHO cell membranes.

Fen-B [12] is a derivative of the Aβ42-raising GSM, fenofibrate [13], coupled to a biotin moiety. Previous report showed that Fen-B directly targeted APP-CTF by photo-crosslinking using recombinant proteins or microsomes under overexpression conditions. However, the possibility of a nonspecific binding of fenofibrate to high concentrations of APP in an artificial condition was not excluded [14,15]. We performed a photoaffinity labeling experiment with Fen-B using microsomes prepared from brains of wild-type mice. We found that endogenous PS1 NTF, but neither of other γ-secretase components (i.e., PS1 CTF, Nicastrin, Aph-1 and Pen-2) nor APP-CTF, was specifically precipitated (Figure 1A). In addition, we detected a biotinylated band of ~30 kDa, corresponding to the molecular weight of PS1 NTF, in the fraction incubated with Fen-B. To further confirm the specificity of labeling of PS1 NTF by Fen-B, membrane fractions of fibroblasts from *Psen1*^*-/-*^/*Psen2*^*-/-*^ double knockout mice (DKO) [16] with or without overexpression of His-tagged PS1 [17] were subjected to PAL. Biotinylated PS1 NTF was specifically precipitated, indicating that PS1 NTF is targeted by Fen-B (Figure 1B). Finally, a specific binding of Fen-B to SPP, another aspartic intramembranous cleaving protease, which shared homology with PS [18], was also observed (Figure 1C). Taken together, we concluded that the *bona fide* molecular target of fenofibrate, in the context of modulation of intramembrane cleavage, are PS1 and SPP, i.e., the enzyme moieties.

**Figure 2:**
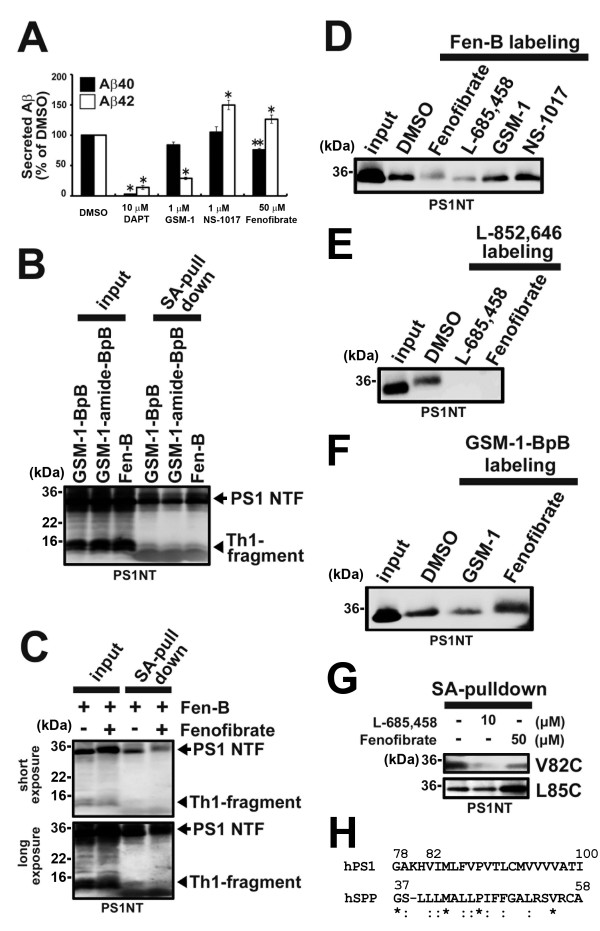
**Cytosolic side of TMD1 forms a fenofibrate binding pocket. (A)** Sensitivity of PS1-Th1 for GSMs. Effect of DAPT (10 μM), GSM-1 (1 μM), NS-1017 (1 μM) and fenofibrate (50 μM) on secreted Aβ from wild-type PS1 containing γ-secretase using DKO cells stably expressing APPNL (n = 3, mean ± SD, *p < 0.01, **p < 0.05 at Student’s t test). **(B)** Thrombin digestion experiments were performed after PAL by GSM-1-BpB (1 μM), GSM-1-amide-BpB (1 μM) and Fen-B (10 μM). Note that cleaved Th1-fragment (arrowhead) was precipitated and detected by anti-PS1 NTF antibody. **(C)** Labeling competition analysis of Fen-B (10 μM) in the presence of fenofibrate (100 μM) using PS1-Th1 microsomes. Upper and lower panels show short and long exposures, respectively. **(D)** Labeling competition analyses were performed with fenofibrate (100 μM), L-685,458 (10 μM), GSM-1 (100 μM) and NS-1017 (100 μM) for the labeling of PS1 NTF by Fen-B (10 μM). **(E)** Labeling competition experiment with L-685,458 (10 μM) and fenofibrate (100 μM) for the labeling of PS1 NTF by L-852,646 (100 nM). **(F)** Labeling competition analysis by GSM-1-BpB (1 μM) in the presence of GSM-1 (100 μM) or fenofibrate (100 μM) using CHO cell microsomes. **(G)** SCAM analyses of microsomes from DKO cells expressing single-Cys mt PS1 containing one Cys at 82 or 85 positions in the presence or absence of indicated compounds. Note that the labeling of V82C was decreased and of L85C was increased by preincubation with fenofibrate. **(H)** Alignment of amino acid residues of PS1 TMD1 (78th to 100th residues) and 37th to 58th residues of SPP, which includes predicted TMD1 (32nd to 54th residues [19]). Asterisks and colons indicate conserved and similar amino acids, respectively.

To narrow down the fenofibrate binding site within PS1 NTF, we employed the limited digestion approach by inserting a thrombin substrate sequence into PS1 [10]. γ-Secretase containing PS1-Th1 mutant, in which thrombin cleavable sequence was inserted between D110 and G111 in the hydrophilic loop 1, harbored γ-secretase activity and was sensitive to fenofibrate (Figure 2A). Eight kDa N-terminal fragment of PS1 NTF generated by thrombin cleavage of PS1-Th1 after Fen-B crosslinking was specifically precipitated in a similar fashion to that by phenylpiperidine-type photoprobes, suggesting that Fen-B also targets to the most N-terminal region of PS1, including TMD1 (Figure 2B and C). We showed that the cytosolic side of TMD1 participates in the catalytic hydrophilic pore [7,11]. To analyze the relationship between the fenofibrate binding site and the catalytic site within TMD1, we employed the cross-competition analysis in Fen-B labeling using different classes of compounds. Labeling of PS1 NTF by Fen-B was diminished by L-685,458, that directly targets the cytosolic side of TMD1 (Figure 2D) [11]. Consistent with this, labeling of PS1 NTF by L-852,646, an L-685,458-based photoprobe, was inhibited by fenofibrate (Figure 2E). In contrast, neither GSM-1 nor NS-1017, which targets the luminal region of TMD1, affected the binding of Fen-B (Figure 2D). Moreover, biotinylation of PS1 NTF by GSM-1-BpB was hardly affected by fenofibrate (Figure 2F), suggesting that the binding site of fenofibrate is distinct from that of GSM-1 within TMD1. We then performed a labeling competition experiment in SCAM [7,11], the latter being a biochemical method to deduce the structure of the membrane protein by position-specific biotinylation and to identify the targeting site of the compound of interest. Preincubation of fenofibrate decreased the biotinylation at Val82, supporting the notion that fenofibrate targets the catalytic site. In contrast, labeling of Leu85 was increased, indicating that fenofibrate evokes a conformational change of the catalytic site in TMD1 (Figure 2G). Intriguingly, Gly37 to Ala58 of SPP, which encompassed the predicted SPP TMD1 (Ile32 to Ser54) [19], showed a substantial homology to primary sequence of N-terminal region of PS1 TMD1 (i.e., Gly78 to Ile100) (Figure 2H), suggesting the possibility that fenofibrate targets to the predicted SPP TMD1. Taken together with the results of chemical biological experiments, the binding site of fenofibrate was estimated to locate around Val82 in TMD1, leading to the conformational change of the catalytic site of γ-secretase.

### Intermediate longer A was secreted by TMD1 mutant PS1

**Figure 3:**
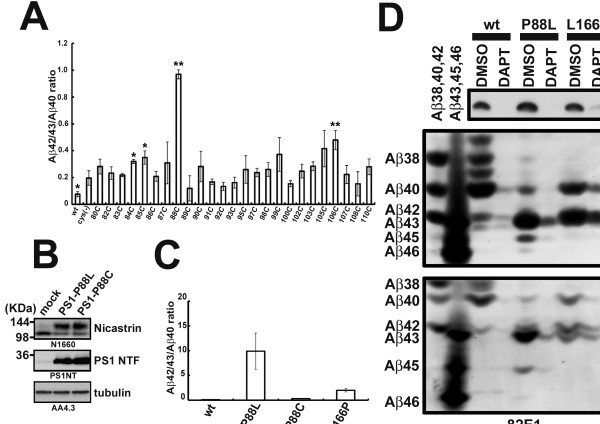
**Pro88 is the critical amino acid residue for the determination of length of secreted Aβ. (A)** Aβ42(43)/Aβ40 ratio in secreted Aβ from DKO cells expressing Single-Cys mt PS1. Conditioned media were analyzed by ELISA (n = 3, mean ± SD, *p < 0.05, **p < 0.01 (Compared with Cys-less) at Student’s t test). **(B)** Immunoblotting analysis of DKO cell lysate expressing PS1 mutant carrying P88C or P88L by indicated antibodies. **(C)** Aβ42(43)/Aβ40 ratio in secreted Aβ from DKO cells in **(B)** measured by ELISA (n = 3, mean ± SD). **(D)** Urea-SDS gel analysis for secreted Aβ from DKO cells expressing wild-type PS1, PS1 with P88L or PS1 with L166P mutation. Conditioned media of cells treated with 10 μM DAPT were also analyzed. Two types of Urea-SDS gels were used to obtain better resolution in the detection of longer Aβ peptides (i.e., Aβ45 and Aβ46).

This finding prompted us to hypothesize that TMD1 is potentially involved in the regulation of the processivity of γ-secretase to generate Aβ42. To address this issue, we screened single cysteine (single-Cys) mutants of TMD1 in cysteine-less PS1 used in SCAM. While cysteine-less PS1 increased Aβ42(43)/Aβ40 ratio compared to that of wild-type PS1-expressing cells, some single-Cys mutants showed further augmentation in Aβ42(43)/Aβ40 ratio (Figure 3A). Among these mutants, P88C mutant showed a dramatic increase in the Aβ42(43)/Aβ40 ratio. To further analyze the functional significance of Pro88 in the processive cleavage, we analyzed two PS1 mutants, P88C and P88L. Surprisingly, P88L mutation caused an increase in the Aβ42(43)/Aβ40 ratio, to a level higher than that with L166P mutant PS1 (Figure 3C and D), one of the most potent pathogenic PS1 mutations [20]. In addition, P88L mutant elicited secretion of the intermediate, longer form of Aβ, i.e. Aβ45 and Aβ46, but neither in the wild-type nor L166P mutant PS1 (Figure 3D). These data strongly implicated TMD1 in the regulation of the C-terminal length of Aβ.

### GSMs and P88L mutation affected the interaction between TMD1 and longer Aβ species

**Figure 4:**
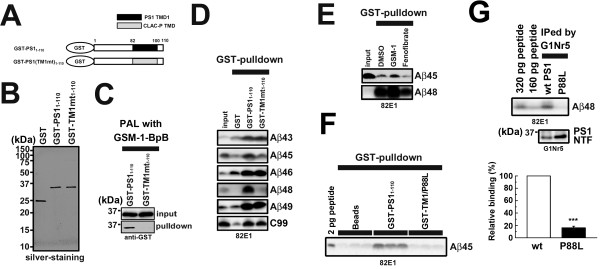
**GSMs and P88L mutation alter interaction of TMD1 of PS1 with Aβ45. (A)** Schematic representation of recombinant proteins used in this study. **(B)** GST and GST-fused recombinant proteins were purified by glutathione sepharose and/or Mono Q column. The purity of purified recombinant proteins was confirmed by silver staining. **(C)** GSM-1-BpB (4 nM) labeling experiment for GST-PS1_1-110_ and TMD1 swapping mutant, GST-TM1mt_1-110_ (0.19 μg). Samples were preincubated with photoprobes on ice for 10 min, and then irradiated for 60 min. **(D)** Pull down assay of GST-fused recombinant proteins mixed with longer Aβ (Aβ43/45/46/48/49) (1 nM). After incubation with glutathione sepharose, all samples were washed and subjected to immunoblot. **(E)** The effects of GSM-1 (25 μM) or fenofibrate (100 μM) on the interaction of GST-PS1_1-110_ with longer Aβ peptides. Recombinant proteins were preincubated with GSMs and then incubated with longer Aβ peptides. Protein complexes were pulled down by glutathione sepharose and analyzed by immunoblot. **(F)** The effect of P88L mutation on the binding of Aβ45 peptide to GST-PS1_1-110_. **(G)** Binding of Aβ48 peptide to native PS1 protein expressed in mammalian cells. Densitometric analysis of relative levels of the bound Aβ48 was shown at the below (n = 3, mean ± SD, ***p <0.001 at Student’s t test).

Our unexpected observation of an abnormal secretion of longer Aβ45 from cells expressing P88L mutant PS1 prompted us to further investigate the functional role of TMD1 during the enzymatic process, especially the processive cleavage. Notably, previous reports indicated that a region between Val82 to Ser132 encompassing TMD1 directly participates in the interaction with γ-secretase substrates (i.e., APP-CTF) *in vitro*[21-23]. Moreover, Aβ46 has been shown to accumulate by DAPT, which inhibits processive cleavage, and to be coimmunoprecipitated with PS1 [23,24]. These results implied the possibility that TMD1 directly recognizes the longer Aβ species during the processive cleavage of γ-secretase. To test this idea, we performed *in vitro* binding assays of various longer Aβ species with purified GST, GST-PS1_1-110_ and GST-TM1mt_1-110_, the latter harboring the TMD1 sequence (i.e., Val82 to Ile100) replaced with a TMD of an unrelated membrane protein, CLAC-P [10,25] (Figure 4A and B). Replacement of TMD1 of PS1 with CLAC-P sequence abolished the γ-secretase activity [17] as well as binding of GSM-1-BpB *in vitro*[10] (Figure 4C), indicating chimeric PS1 with CLAC sequence has a distinct property. Then synthetic Aβ peptides were coincubated with recombinant proteins and pulled down by glutathione sepharose (Figure 4D). In this condition, we detected specific binding of recombinant C99-FLAG to GST-PS1_1-110_, as previously reported by Annaert et al [21]. In addition, we detected binding of all synthetic longer Aβ peptides (Aβ49, Aβ48, Aβ46, Aβ45, Aβ43) with GST-PS1_1-110_, suggesting that TMD1 of PS1 directly binds to longer Aβ peptides, and that the cytoplasmic domain of APP is not involved in this binding. Unexpectedly, GST-TM1mt_1-110_ was capable of interacting with C99-FLAG as well as with peptides belonging to the Aβ40 production line (Aβ49, Aβ46 and Aβ43). However, the binding of Aβ species of the Aβ42 production line (i.e., Aβ48 and Aβ45) was significantly reduced by swapping the TMD1 sequence (Figure 4D). We further analyzed the effect of TMD1-targeting GSMs as well as P88L mutation on the binding of Aβ45 and Aβ48 to TMD1. Intriguingly, GSM-1 augmented the interaction of TMD1 with Aβ45 as well as Aβ48, whereas it was reduced by fenofibrate (Figure 4E). In addition, introduction of P88L mutation in GST PS1-_1-110_ decreased the pull down of Aβ45 (Figure 4F). Finally, we observed a specific binding of Aβ48 with native PS1 protein expressed in the mammalian cells, and this interaction was almost diminished by the P88L mutation (Figure 4G). We did not observe specific binding of Aβ45 with PS1 holoprotein expressed in mammalian cells, presumably due to weak binding of Aβ45 to PS1 protein. Nevertheless, these data suggest that the processivity of the γ-secretase for Aβ42 production is defined by the tenacity of interaction between TMD1 and longer Aβ, which may determine the retention of the substrate in the catalytic site. Collectively, we uncovered the significant function of TMD1 of PS1 as a binding site for the longer Aβ species, especially Aβ45 and Aβ48, during the processive cleavage of the Aβ42 production line, and the effects of GSMs on Aβ42 production by changing the affinity between TMD1 and the longer Aβ peptides.

## Discussion

Understanding the molecular mechanism of the processive cleavage by γ-secretase is critical to the development of effective GSMs. We previously reported that phenylpiperidine-type GSMs are bound to TMD1 of PS1 [10]. Here, we further showed that fenofibrate, an Aβ42-raising GSM, also directly targets TMD1, while Fen-B was reported as APP-targeting photoprobe [12]. Recently, some papers reported that large amount of Aβ42 or C99 forms aggregates that cause non-specific binding to GSMs [14,15]. Therefore, we have used brain microsomes obtained from wild-type mouse for the photo-crosslinking experiment.

Scissile bonds for processive cleavage by γ-secretase have hypothetically been mapped on different surfaces in the α-helical model of APP TMD [26]. This raises the possibility that the distinct processive cleavages by γ-secretase, i.e., those leading to production of Aβ49-46-43-40 or Aβ48-45-42-38, are determined by the recognition of one or the other of the specific helical surfaces. However, the domain on γ-secretase that recognizes the helical surface on the substrate is yet to be identified. It has previously been suggested that TMD1 of PS1 is involved in the binding of APP-CTF, a direct substrate of γ-secretase [21,22]. Here we found that longer Aβ peptides that are generated as intermediate products in the Aβ42 production line (i.e., Aβ45 and Aβ48), which also are direct substrates for the processive cleavage, retain the capacity to interact with TMD1 of PS1. It is highly likely that the “gripping tenacity” of the substrate binding site facing the catalytic pore would determine the processivity of Aβ48 and Aβ45 on the Aβ42 production line, which can be modulated by small compounds. Consistently, Okochi et al. have recently reported that Aβ42 is bound to the γ-secretase complex [27] and the binding was modulated by GSMs, although they have not identified the binding site of Aβ within the enzyme complex. Thus, we propose that TMD1 of PS1 functions as a binding site of longer Aβ species for γ-secretase during the processive cleavage, which specifically determines the efficiency of the processive cleavage of the Aβ42 production line. Structural analyses suggested that the catalytic cavities of rhomboid protease [28], another intramembrane-cleaving enzyme, or those of FlaK [29] and PSH [30], archaeal GxGD proteases, are unable to accommodate all the amino acid residues of the transmembrane sequence of the substrates. This suggests that a major part of the TMD of substrates remains within the membrane and is gripped by enzymes to incorporate the cleavage site into the intramembrane catalytic site during proteolysis. While the precise structure of human PS1 still remains unclear, our SCAM results on PS1 [11], as well as the recently reported x-ray crystal structure of PSH [30], the latter being composed of 9-transmembrane domains similarly to human PS1, altogether suggested that TMD1 locates in proximity to the catalytic aspartate in TMD7. The results of these structural analyses also support our notion that TMD1 functions as a substrate binding domain during the processive cleavage by γ-secretase.

**Figure 5:**
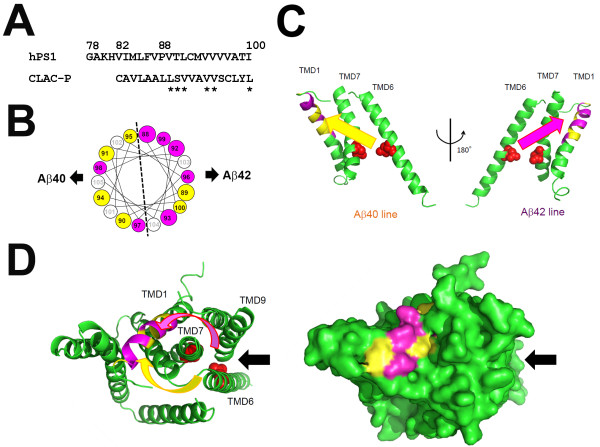
**Hypothetical model of the role of PS1 TMD1 in Aβ42 generation. (A and B)** Alignment of amino acid residues of the human PS1 TMD1 and CLAC-P. In the swap mutant PS1, 82nd to 100th residues were replaced with CLAC-P TMD. Residues with similar side chains are labeled with asterisks in **(A)** and as yellow circles in **(B). (C)** Modified structural model of PS1 based on PSH structure kindly provided from Dr. Y. Shi [30]. Side views of the ribbon model of TMD1, 6 and 7 generated by Pymol are shown. Yellow and purple residues correspond to those in **(B)**. Possible approaches to the catalytic aspartates (red spheres) for Aβ40 and Aβ42 production lines are shown by yellow and purple arrows, respectively. TMD1 grips the substrate to prevent its liberation during the processive cleavage. Helix-helix interaction of longer Aβ with TMD1 determines the major production line (i.e., Aβ40 or Aβ42) of processive cleavage. **(D)** Top views of the whole PS1 model (left; ribbon diagram; right, surface model) generated by Pymol are shown. Putative substrate entry site [30] is shown as black arrows.

TMD1 of PS1 bound not only to longer Aβ peptides of the Aβ42 production line, but to those of Aβ40 line (Figure 4D). P88L mutation in TMD1 of PS1 caused an increased secretion of not only Aβ45, but also Aβ43 and Aβ46 (Figure 3F), suggesting that the interaction between TMD1 and longer Aβ species is also critical for the processivity in the Aβ40 production line. Intriguingly, swapping TMD1 sequence of PS1 with that of CLAC-P, an unrelated membrane protein, did not affect the binding of Aβ43, Aβ46 and Aβ49 peptides. In the helical net diagram, similar side chains at the luminal side of PS1 TMD1 and CLAC-P TMD comprised an interface on the α-helical model only in one side (Figure 5A and B) [31]. Thus, one helical surface of TMD1 is involved in the binding of longer Aβ species in the Aβ40 production line, whereas the other surface specifically interacts with those in the Aβ42 production line in the PSH-based PS1 model (Figure 5C). Pharmacological and chemical biological studies suggest that the substrate enters the catalytic site via the initial substrate binding site, in which TMD2, 6 and 9 are involved [17]. Especially, TMD6 and 9 have been implicated in the lateral entry of the substrate from the crystal structure of PSH [30]. However, helical peptide-type GSIs that target the initial substrate binding site equally inhibited the production of Aβ40 or Aβ42 [32,33]. Thus, we hypothesize that C99 or longer Aβ is gripped by TMD1 after the lateral entry (Figure 5D). Structural model also suggested that residues related to the Aβ42 production line in the luminal side of TMD1 are located on the surface of PS1 polypeptide, which might be targeted by GSM-1 [10]. In fact, several side chain interactions have been identified in TMD1 and the neighboring TMDs [30]. However, we have previously suggested a piston-like vertical movement of TMD1 by SCAM [34], supporting our view that dynamic conformational changes would take place during the catalytic reaction. Intriguingly, the reason why the efficiency of Aβ42 production is always lower than that of Aβ40 in any cell types [35] has been unknown. Considering the positions of the first ϵ-cleavage sites located on the opposite sides of the helical surfaces predicted for the Aβ40 and Aβ42 production lines, it is tempting to speculate that an approach of the substrate from an unfavored direction (pink arrow, Figure 5) to the catalytic site in terms of stereochemistry might explain the lower efficiency of cleavage in the Aβ42 production line (Figure 5C and D). However, the other TMDs might be also involved in the recognition of Aβ42, and GSMs could allosterically affect these other regions including initial substrate binding site in PS1. Biochemical analysis to identify the other binding domain for longer Aβ peptides belonging to the Aβ40 production line would provide further molecular insights regarding the mechanism of action of GSMs. Also structural analysis of mammalian PS1 carrying P88L mutation might unveil further mechanistic role of TMD1 in the trimming process by γ-secretase. In sum, we revealed that TMD1, a previously identified target region of GSMs, participates in the Aβ42 generation as a binding site that docks longer Aβ species as intermediate substrates for γ-secretase. Our observations may shed light on the molecular mechanism of the processive cleavage by γ-secretase, contributing to the development of potent and selective Aβ42-lowering compounds for AD.

## Conclusions

Fenofibrate directly bound to TMD1 of PS1 to induce the conformational changes in the catalytic site of the γ-secretase. P88L mutation in TMD1 caused an aberrant secretion of longer Aβ polypeptides (i.e., Aβ45 or Aβ46), indicating that TMD1 is involved in the regulation of C-terminal length of Aβ. Finally, we found that TMD1 contains a binding site for the longer Aβ species, and GSMs affect Aβ42 production by changing the affinity between TMD1 and longer Aβ. Our results suggest that TMD1 functions as a substrate binding domain during the processive cleavage by γ-secretase.

## Methods

### Compounds, peptides and antibodies

GSM-1, GSM-1-BpB, NS-1017, GSM-1-amide-BpB, Fen-B and DAPT were synthesized as described [10,12,36]. L-685,458 and fenofibrate were purchased from Bachem and SIGMA, respectively. L-852,646 [37] was kindly provided from Dr. Y. Li (Sloan-Kettering Cancer Center). Synthetic longer Aβ peptides (i.e., β-amyloid (1-43, #23573), (1-45, #61956-01), (1-46, #62076-01), (1-48, #61965-01), (1-49, #61963-01) were purchased from Anaspec. Aβ (1-40) (#4307-v) and Aβ (1-42) (#4349-v) peptides were purchased from Peptide institute. The rabbit polyclonal antibodies anti-PS1 NTF (G1Nr5), anti-PS1 CTF (G1L3) and anti-Pen-2 (PNT3) were raised as described [38-40]. Anti-PS1 NTF (PS1NT) [41] and anti-SPP (SPPc) [42] were kindly gifted from Drs. G. Thinakaran (The University of Chicago) and T. Golde (University of Florida). Anti-nicastrin N1660 (SIGMA), anti-APP CTF (Immuno-Biological Laboratories), anti-Aph-1aL O2C2 (Covance), anti-human Aβ 82E1 (Immuno-Biological Laboratories) and anti-biotin (Bethyl) were purchased from indicated vendors. The monoclonal antibody anti-α-tubulin AA4.3 developed by Dr. C. Walsh was obtained from the Developmental Studies Hybridoma Bank developed under the auspices of the NICHD, National Institutes of Health, and maintained by The University of Iowa, Department of Biology, Iowa City, IA.

### Plasmid construction, cell culture manipulation and cell based assay

cDNAs encoding PS1 and APP carrying Swedish mutation (APPNL) were inserted into pMXs-puro [43]. cDNAs encoding mutant PS1 were generated by long PCR-based QuikChange^TM^ strategy (Stratagene). To produce recombinant proteins, cDNAs encoding PS1 were cloned into pGEX-6P-1 vector (GE healthcare) [10]. Maintenance of cultured cells, transfection, retroviral infection, two-site enzyme-linked immunosorbent assay (ELISA), or immunoblotting using Urea/SDS-PAGE gel system as described [10,39,44,45].

### Photoaffinity labeling and SCAM experiments

Preparation of samples for photoaffinity labeling experiments [46] was performed as follows. Brains of C57J/B6 mouse (3-5 month age) or cultured cells were homogenized with homogenize buffer (20 mM HEPES (pH 7.0), 140 mM KCl, 250 mM sucrose, 0.5 mM diisopropyl fluorophosphate, 0.5 mM phenylmethylsulfonyl fluoride, 1 μg/ml tosyllysine chloromethyl ketone, 1 μg/ml antipain, 1 μg/ml leupeptin, 10 μg/ml phosphoramidon, 5 mM EDTA, 1 mM EGTA) using Potter-Elvehjem Tissue Grinder (Wheaton), and membrane fractions were collected by ultracentrifugation at 100,000 × g (Beckman) [10]. PAL experiments utilizing avidin-biotin catch principle [47] and thrombin digestion experiments after PAL were performed as previously described [10]. Briefly, after resuspension of the microsome in the homogenize buffer by 25G needle with syringe, protein content was measured by BCA assay (Thermo Fisher Scientific). 1 mL of microsome-containing solution (1 mg/ml protein) was preincubated with compounds for 30 min on ice. Then photoprobes were added and incubated for 10 min on ice under the dark condition. UV irradiation (352 nm) was performed on ice for 1 hr with a UV lamp (Model XX-15BLB, UVP). The approximate distance from UV lamp to the samples was 10 cm. The biotinylated proteins were precipitated by streptavidin sepharose (GE healthcare) in 1% SDS containing homogenization buffer. For SCAM, all methanethiosulfonate reagents (Toronto Research Chemicals) were dissolved in dimethyl sulfoxide at 200 mM prior to use or stored at 80 degree until use. The methods for SCAM and competition experiments using biotinylaminoethyl methanethiosulfonate have been described in detail before [10,11]. Briefly, stable DKO cells expressing cysteine mutant PS1 were grown on two 15-cm dishes per single analysis. Cells were scraped in PBS and resuspended in 2 ml of SCAM homogenization buffer (10 mM HEPES (pH 7.4), 150 mM NaCl, 10% glycerol, Complete protease inhibitor cocktail (Roche Biochemicals)). Cells were disrupted by a Polytron homogenizer (Hitachi), and nuclei and large cell debris were pelleted by centrifugation at 1,500 × g for 10 min. The postnuclear supernatants were centrifuged, and the resultant microsomal pellets were resuspended in 0.2 ml of PBS in a syringe, and 0.1 mM biotinylaminoethyl methanethiosulfonate was added to this fraction. After 30 min incubation at 4 degree, microsomes were centrifuged twice to wash out. The resultant pellets resuspended in 1% SDS/PBS were incubated with the streptavidin sepharose overnight and analyzed as in the intact cell biotinylation experiment. In PAL or SCAM experiments, we loaded 1.5 and 20% of samples as “input” and “bound”, respectively, in all immunoblot analyses.

### Protein purification and binding assay

GST-fusion recombinant proteins were expressed in *E. Coli* (BL21 DE3) (Novagen) and purified by two step procedures using glutathione sepharose and mono Q columns (GE Healthcare) as manufacturer’s instruction. All recombinant proteins were finally diluted with recombinant protein preparation buffer (10 mM HEPES (pH 7.4), 150 mM NaCl, 0.25% CHAPSO). C99-FLAG was purified from Sf9 cells infected with recombinant baculovirus encoding C99-FLAG and diluted at 1 μg/ml in recombinant protein preparation buffer. To perform binding assay, 0.5 μg of GST-fusion recombinant proteins were mixed at 1 μg of C99-FLAG, 1 (for Aβ43, Aβ45, Aβ46, Aβ48 and Aβ49) or 10 (for Aβ40 and Aβ42) nM of synthetic Aβ in 1 ml of recombinant protein preparation buffer, and incubated at 4 degree overnight. After addition of glutathione sepharose, samples were then washed with the buffer and precipitates were eluted by boiling in sample buffer. For binding assay using native PS1 protein, PS1 or P88L mutant PS1 was expressed in DKO cells and solubilized in 10 mM HEPES buffer containing 1% CHAPSO. After addition of 2 ng of Aβ48 peptide, the solubilized fraction was incubated with anti-PS1 antibody G1Nr5 at 4 degree overnight. PS1-Aβ48 complex was then immunoprecipitated using Protein G sepharose 4 Fast Flow (GE Healthcare). Subsequently eluates (i.e., proteins bound to GST-fusion recombinant proteins or native PS1 proteins) were analyzed by immunoblotting. We loaded 0.75 and 20% of samples as “input” and “bound”, respectively, in immunoblot of all pull down assay unless the amount of loaded proteins was otherwise indicated.

## Abbreviations

AD: Alzheimer disease; CTF: Carboxyl-terminal fragment; DKO: *Psen1*/*Psen2* double knockout mouse immortalized fibroblasts; GSM: γ-secretase modulator; NTF: Amino-terminal fragment; mt: Mutant; PS: Presenilin; SCAM: Substituted cysteine accessibility method; TMD: Transmembrane domain.

## Competing interests

The authors declare that they have no competing interests.

## Authors’ contributions

YO and TT designed the research. YO, AT, SO and TT performed biochemical experiments. NS, TH, SY and TF synthesized the compounds. YO, TT and TI wrote the paper. All authors read and approved the final manuscript.
